# Supplementary material for: G4Beacon: An In Vivo G4 Prediction Method Using Chromatin and Sequence Information
Source: Biomolecules. 2023 Feb 3;13(2):292. doi: 10.3390/biom13020292 (PMC9953394; doi:10.3390/biom13020292)
Supplement: Supplementary file 1 [file biomolecules-13-00292-s001.zip › biomolecules-2123990-supplementary.pdf]

# Dataflow of G4Beacon

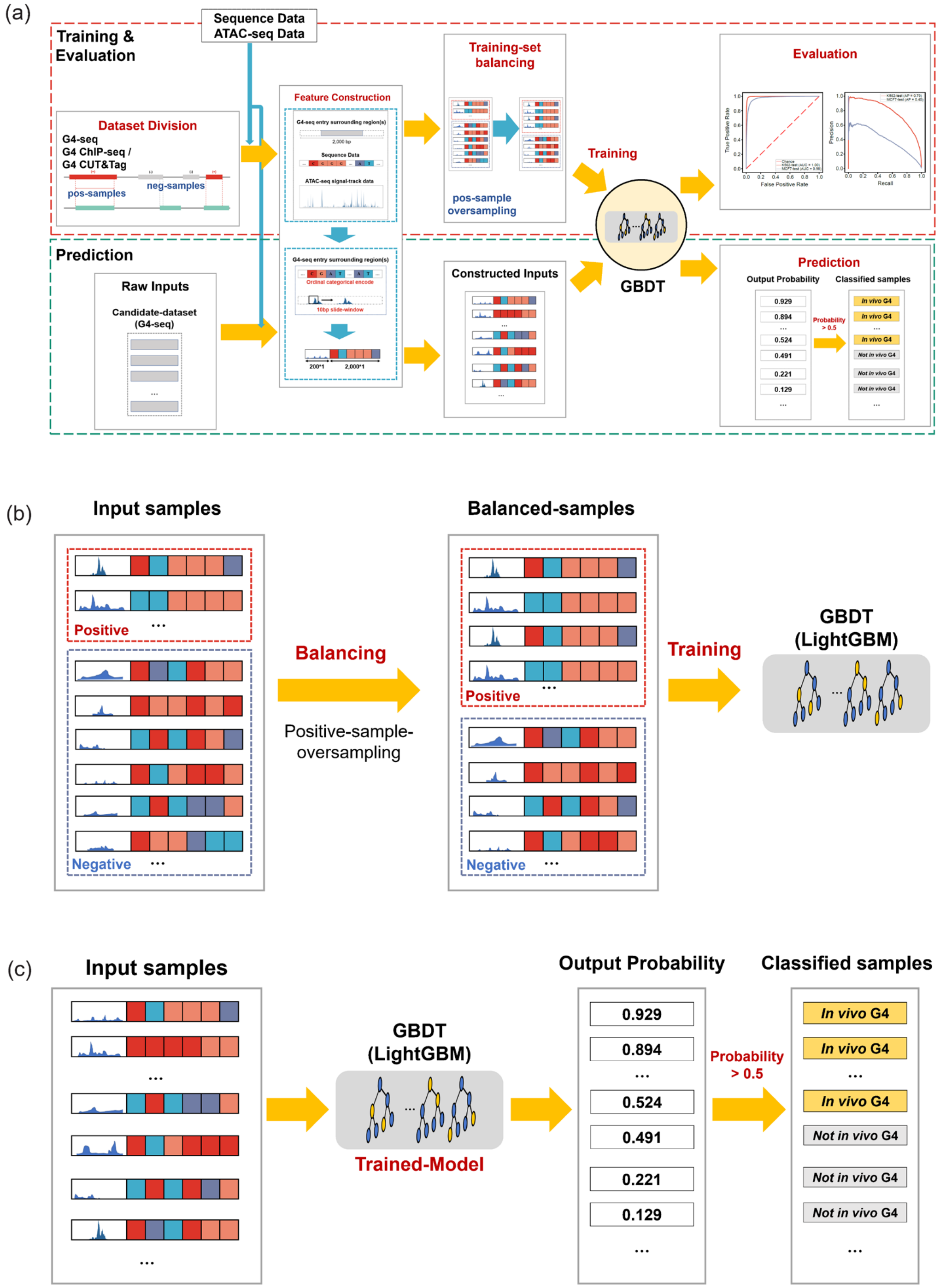


**Figure S1.** Workflow of G4Beacon. (a) Overview of G4Beacon training and prediction workflow. In both the training and the prediction workflow, the feature construction is applied on all samples of an input dataset. Differences between these two workflows are that the training inputs should be labeled as positive or negative samples, and pos–neg balancing is employed before the model training. (b) The pos–neg balancing step of the training set. The over-sampling method is used and the processed dataset has the pos:neg ratio of 1:1. (c) The dataflow of prediction after feature construction. G4Beacon will output the probability of a sample being an active G4 and the typical threshold is 0.5.

## The Overlapping Threshold of Dataset Division

In this research, we used G4-seq as a candidate dataset and overlapped the G4-seq entries with G4 ChIP-seq/G4 CUT&Tag peaks for positive/negative sample division. Unlike the default option of using overlapping threshold=1bp, we employed a stricter threshold: 10% length of the G4 ChIP-seq/G4 CUT&Tag peaks. We considered that this threshold allowed the overlapped regions to be at least one G4 motif length, which might be helpful to mitigate the false positive problem. We compare the differences in the ChIP-seq signal values between the two groups of G4-seq entries that overlapped with in vivo G4s but had overlapping rates ≥10% or <10%, and we found that the ≥10% group had significant G4 ChIP-seq signal values (Wilcoxon test p-value < 2.2e-16).

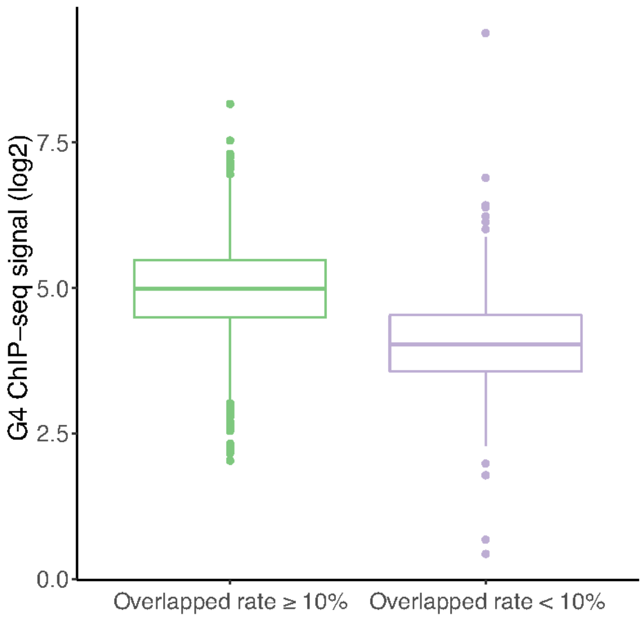


**Figure S2.** The boxplots of G4 ChIP-seq signal of G4-seq entries with overlapped rate ≥ 10% and < 10% on K562 dataset (Wilcoxon test p-value < 2.2e-16).

# Balancing Strategy Comparison

We compared different balancing stratigies for the training set, including under-sampling, over-sampling, and mix-sampling. The under-sampling method downsamples the negative set to be the same size as the positive set, while over-sampling upsamples the positive samples to match the size of the negative set. The mix-sampling employs both under- and over-sampling on the dataset. For example, the **'o0.1_u0.5'** in the following figure and the table indicates that we "upsample the positive set to meet the size of 10% of the negative set size and then downsample the negative set to make the pos:neg ratio = 0.5". We compared these methods in the one-cell-line experiment of HepG2. According to the results, the over-sampling and mix-sampling strategies performed better, and we chose the over-sampling strategy as our balancing method because it does not rely on any empirical parameters.

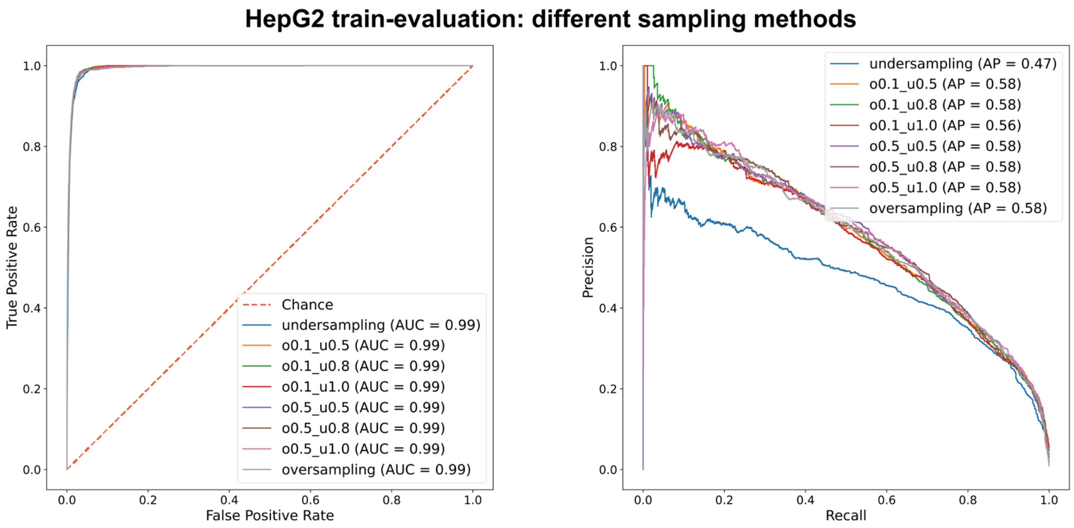


**Figure S3.** ROC/PRC results of the HepG2 cell line train–evaluation employing different sampling methods.

**Table S1.** HepG2 train–evaluation results of the lightGBM models employing different sampling methods.

| | Accuracy | Precision | Recall | F1-Score | AUROC | AP |
|---|---|---|---|---|---|---|
| **Under-sampling** | 0.96 | 0.12 | 0.98 | 0.22 | 0.99 | 0.47 |
| **o0.1_u0.5** | 0.99 | 0.38 | 0.79 | 0.51 | 0.99 | 0.58 |
| **o0.1_u0.8** | 0.99 | 0.32 | 0.85 | 0.47 | 0.99 | 0.58 |
| **o0.1_u1.0** | 0.99 | 0.30 | 0.87 | 0.44 | 0.99 | 0.56 |
| **o0.5_u0.5** | 0.99 | 0.63 | 0.49 | 0.55 | 0.99 | 0.58 |
| **o0.5_u0.8** | 0.99 | 0.55 | 0.60 | 0.57 | 0.99 | 0.58 |
| **o0.5_u1.0** | 0.99 | 0.52 | 0.66 | 0.58 | 0.99 | 0.58 |
| **Over-sampling** | 0.99 | 0.62 | 0.48 | 0.54 | 0.99 | 0.58 |

## Machine Learning Method Selection

To find the best model for the in vivo G4 prediction problem, we selected four candidate machine learning methods, namely Logistic Regression (Logit), Decision Tree (DT), Random Forest (RF), and GBDT (LightGBM implement version), and compared their performance in the K562/HepG2/MCF7 one-cell-line training–evaluation experiment. All the methods employed the default hyperparameters and settings. By comparing the results, we found that LightGBM was the most robust model for our problem.

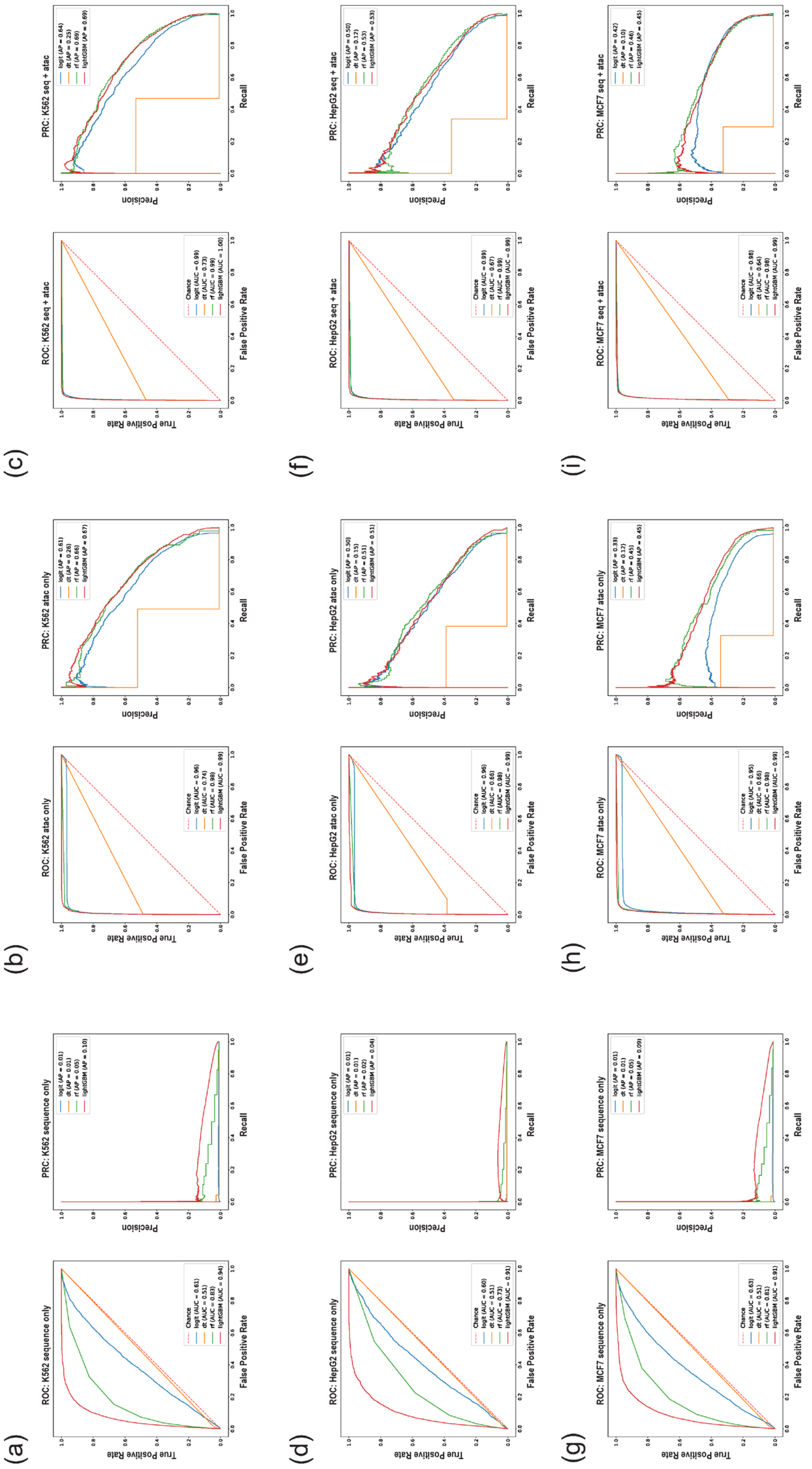


**Figure S4.** ROC/PRC of 4 candidate models (Logistic Regression, Decision Tree, Random Forest, and LightGBM) employed for in vivo G4 prediction with training set and test set derived from the same cell line (K562, HepG2, or MCF7). (a-c) The ROC/PRC of K562 cell line. (d-f) The ROC/PRC of HepG2 cell line. (g-i) The ROC/PRC of MCF7 cell line.

**Table S2.** K562 train–evaluation results of different models employing default parameters.

| | Accuracy | Precision | Recall | F1-score | AUROC | AP |
|---|---|---|---|---|---|---|
| **Logit (seq)** | 0.72 | 0.01 | 0.39 | 0.02 | 0.61 | 0.01 |
| **Logit (ATAC)** | 0.92 | 0.09 | 0.96 | 0.16 | 0.96 | 0.61 |
| **Logit (ATAC+seq)** | 0.98 | 0.27 | 0.93 | 0.42 | 0.99 | 0.64 |
| **DT (seq)** | 0.98 | 0.03 | 0.04 | 0.03 | 0.51 | 0.01 |
| **DT (ATAC)** | 0.99 | 0.52 | 0.49 | 0.51 | 0.74 | 0.26 |
| **DT (ATAC+seq)** | 0.99 | 0.53 | 0.47 | 0.50 | 0.73 | 0.25 |
| **RF (seq)** | 0.99 | NaN | NaN | NaN | 0.82 | 0.05 |
| **RF (ATAC)** | 0.99 | 0.67 | 0.57 | 0.62 | 0.98 | 0.66 |
| **RF (ATAC+seq)** | 0.99 | 0.75 | 0.52 | 0.61 | 0.99 | 0.69 |
| **lightGBM (seq)** | 0.94 | 0.09 | 0.63 | 0.16 | 0.93 | 0.11 |
| **lightGBM (ATAC)** | 0.99 | 0.40 | 0.85 | 0.55 | 0.99 | 0.67 |
| **lightGBM (ATAC+seq)** | 0.99 | 0.47 | 0.83 | 0.60 | 0.99 | 0.69 |

**seq:** sequence-only; **ATAC:** ATAC-only; **seq+ATAC:** sequence–ATAC combined.

**Logit:** Logistic Regression; **DT:** Decision Tree; **RF:** Random Forest.

**Table S3.** HepG2 train–evaluation results of different models employing default parameters.

| | Accuracy | Precision | Recall | F1-score | AUROC | AP |
|---|---|---|---|---|---|---|
| **Logit (seq)** | 0.75 | 0.01 | 0.34 | 0.02 | 0.60 | 0.01 |
| **Logit (ATAC)** | 0.90 | 0.05 | 0.96 | 0.10 | 0.96 | 0.50 |
| **Logit (ATAC+seq)** | 0.98 | 0.18 | 0.93 | 0.31 | 0.99 | 0.50 |
| **DT (seq)** | 0.99 | 0.01 | 0.02 | 0.02 | 0.51 | 0.01 |
| **DT (ATAC)** | 0.99 | 0.39 | 0.38 | 0.39 | 0.66 | 0.15 |
| **DT (ATAC+seq)** | 0.99 | 0.36 | 0.34 | 0.35 | 0.67 | 0.12 |
| **RF (seq)** | 0.99 | NaN | NaN | NaN | 0.73 | 0.02 |
| **RF (ATAC)** | 0.99 | 0.59 | 0.44 | 0.51 | 0.98 | 0.51 |
| **RF (ATAC+seq)** | 0.99 | 0.69 | 0.31 | 0.42 | 0.99 | 0.53 |
| **lightGBM (seq)** | 0.95 | 0.06 | 0.44 | 0.10 | 0.91 | 0.05 |
| **lightGBM (ATAC)** | 0.99 | 0.30 | 0.81 | 0.43 | 0.99 | 0.51 |
| **lightGBM (ATAC+seq)** | 0.99 | 0.35 | 0.77 | 0.48 | 0.99 | 0.53 |

**Table S4.** MCF7 train–evaluation results of different models employing default parameters.

| | Accuracy | Precision | Recall | F1-Score | AUROC | AP |
|---|---|---|---|---|---|---|
| **Logit (seq)** | 0.71 | 0.02 | 0.44 | 0.03 | 0.64 | 0.01 |
| **Logit (ATAC)** | 0.94 | 0.14 | 0.94 | 0.24 | 0.95 | 0.34 |
| **Logit (ATAC+seq)** | 0.97 | 0.24 | 0.93 | 0.39 | 0.98 | 0.42 |
| **DT (seq)** | 0.98 | 0.03 | 0.04 | 0.03 | 0.51 | 0.01 |
| **DT (ATAC)** | 0.99 | 0.34 | 0.33 | 0.33 | 0.66 | 0.12 |
| **DT (ATAC+seq)** | 0.99 | 0.33 | 0.29 | 0.31 | 0.64 | 0.10 |
| **RF (seq)** | 0.99 | NaN | NaN | NaN | 0.81 | 0.05 |
| **RF (ATAC)** | 0.99 | 0.54 | 0.36 | 0.44 | 0.98 | 0.45 |
| **RF (ATAC+seq)** | 0.99 | 0.60 | 0.20 | 0.30 | 0.99 | 0.46 |
| **lightGBM (seq)** | 0.93 | 0.08 | 0.61 | 0.15 | 0.91 | 0.09 |
| **lightGBM (ATAC)** | 0.98 | 0.28 | 0.87 | 0.43 | 0.99 | 0.45 |
| **lightGBM (ATAC+seq)** | 0.98 | 0.31 | 0.86 | 0.46 | 0.99 | 0.45 |

# Hyperparameter Selection

We utilized a grid search method for the hyperparameter selection of LightGBM. Three hyperparameters, i.e., learning_rate (learning rates) with the candidate set of [0.01, 0.1, 0.5, 1.0], n_estimators (numbers of basic estimators) with the candidate set of [100, 500, 800, 1000, 1500, 1800, 200], and n_leaves (numbers of leaves for basic estimators) with the candidate set of [15, 31, 63, 127, 255], were combined and used for configurations. We employed a two-fold cross-validation experiment on the HepG2 cell line dataset, which was basically the same as the one-cell-line experiment that we described in Section 2.5 in the manuscript. As a result, we selected {learning_rate=0.1, n_estimators=1000 and n_leaves=31} as the default configuration for G4Beacon.

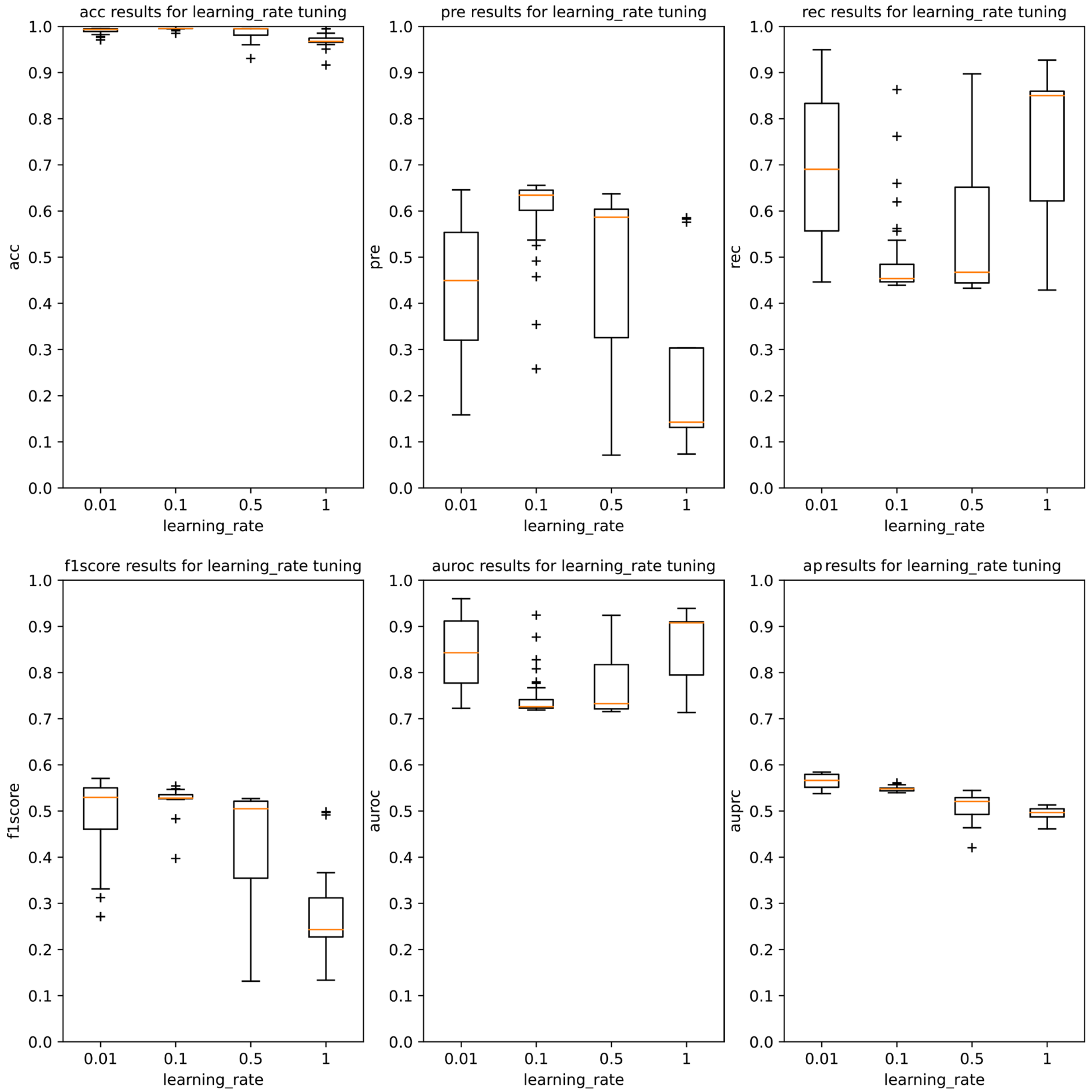


**Figure S5.** Boxplots of the criteria of acc (Accuracy), pre (Precision), rec (Recall), F1-score, AUROC, and AP of a 2-fold cross-validation with different learning rates, on the HepG2 cell line dataset.

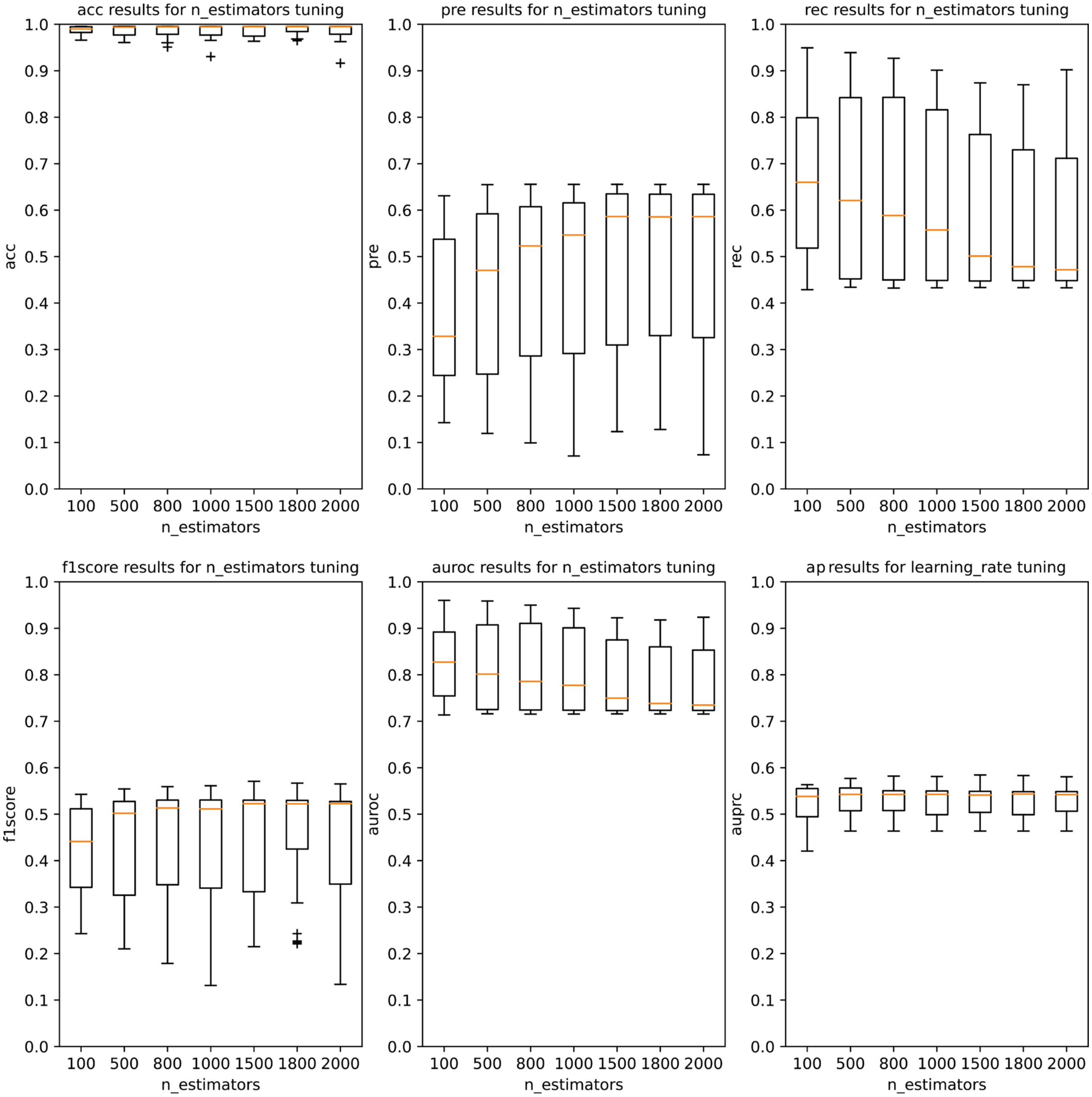


**Figure S6.** Boxplots of the criteria of acc (Accuracy), pre (Precision), rec (Recall), F1-score, AUROC, and AP of a 2-fold cross-validation with different estimator numbers, on the HepG2 cell line dataset.

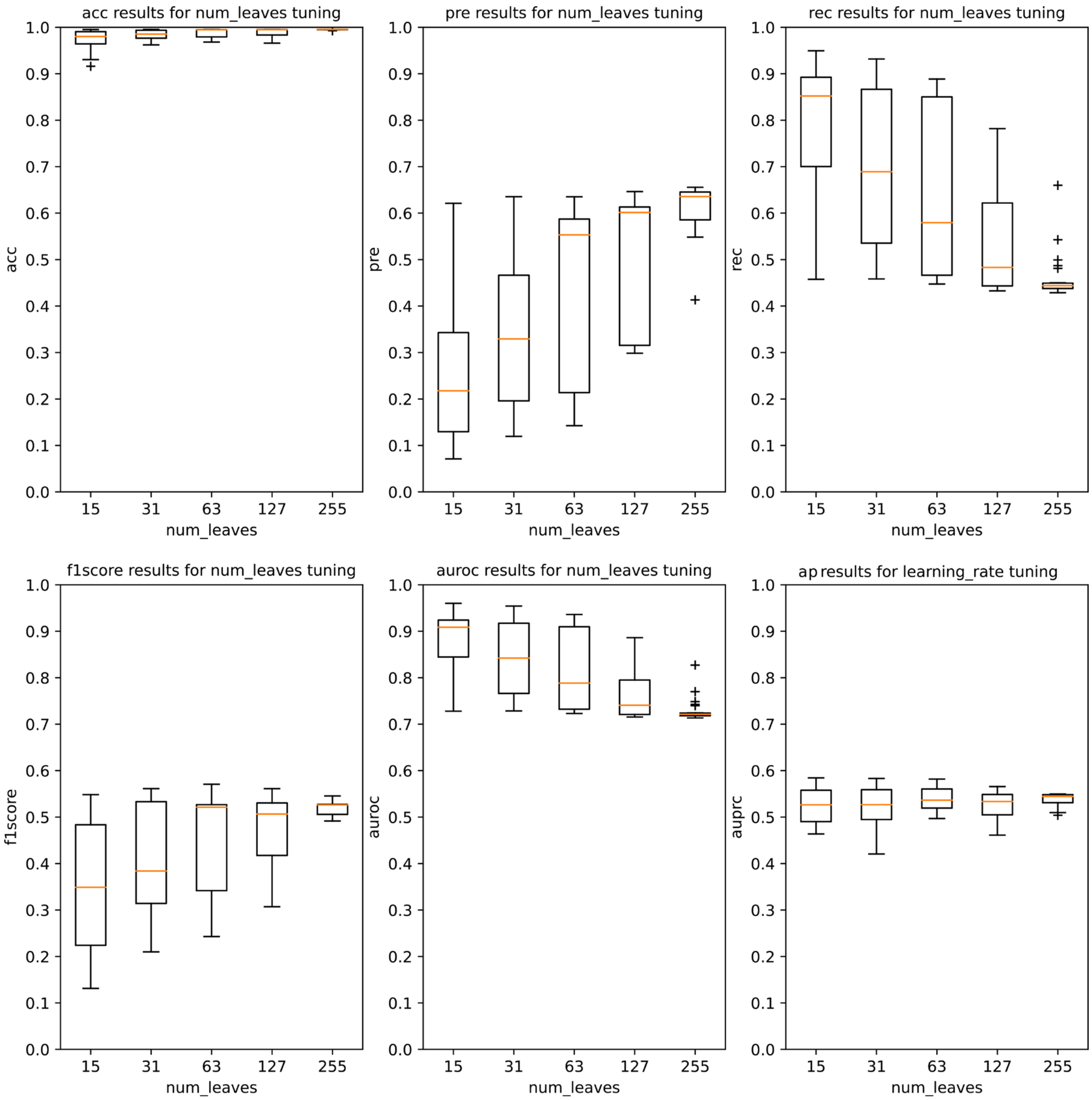


**Figure S7.** Boxplots of the criteria of acc (Accuracy), pre (Precision), rec (Recall), F1-score, AUROC, and AP of a 2-fold cross-validation with different leaf number thresholds of a base estimator, on the HepG2 cell line dataset.

# Supplementary Figures of the Histone Modification States

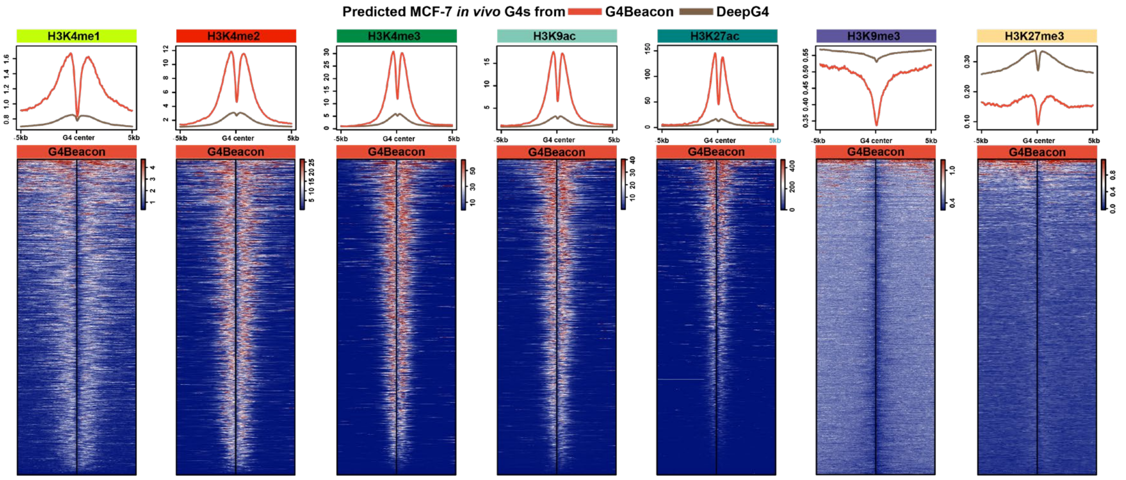


**Figure S8.** Results of the histone modification state analysis on G4Beacon and DeepG4. The line charts and heatmaps of histone modification states around MCF7 active G4s derived from G4Beacon and DeepG4, with the probability threshold of 0.5.

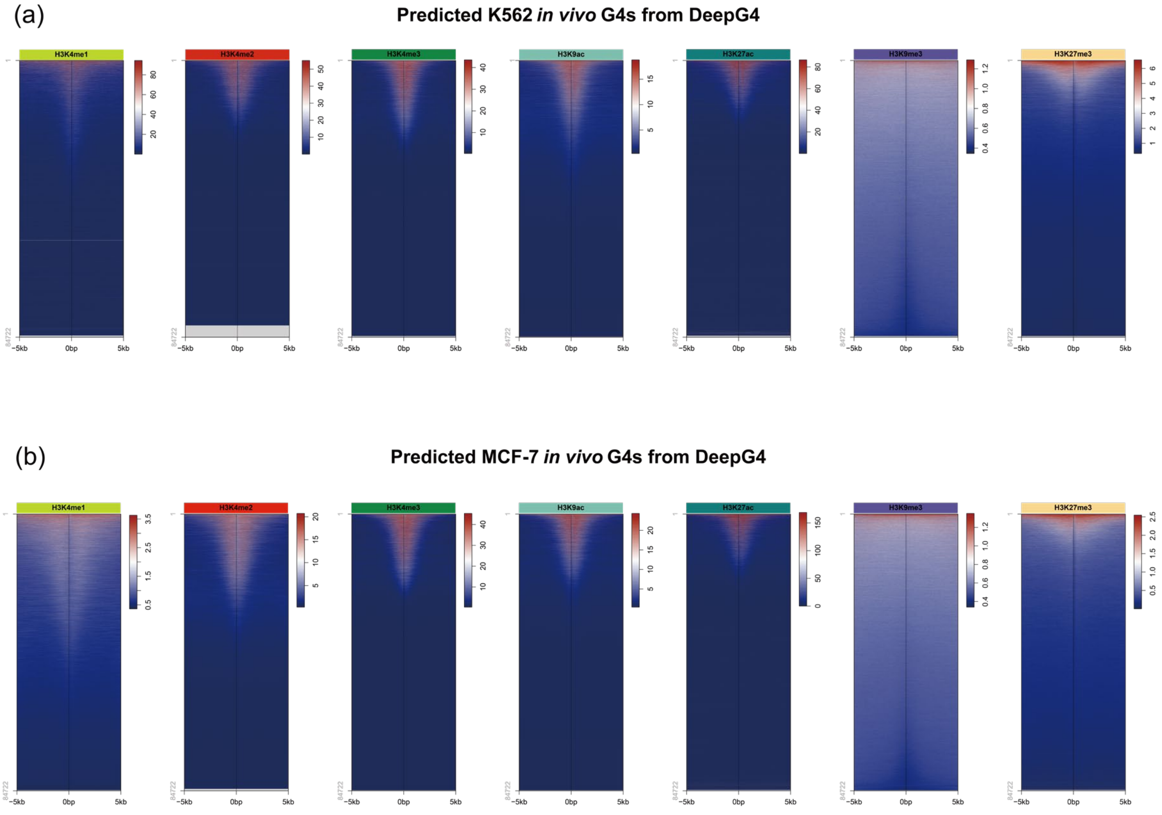


**Supplementary Figure S9.** The heatmaps of histone modification states around active G4s derived from DeepG4. (a) Heatmaps of histone modification states around K562 active G4s predicted by DeepG4. (b) Heatmaps of histone modification states around MCF7 active G4s predicted by DeepG4.

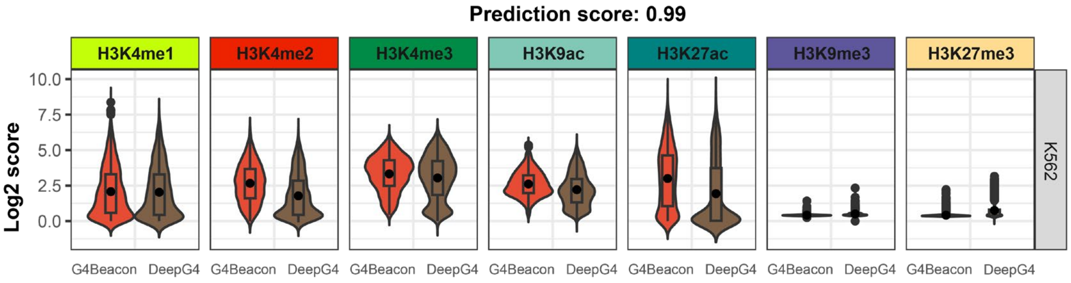


**Figure S10** The boxplots of histone modification state scores of K562 active G4s derived from G4Beacon and DeepG4, with the probability threshold of 0.99.

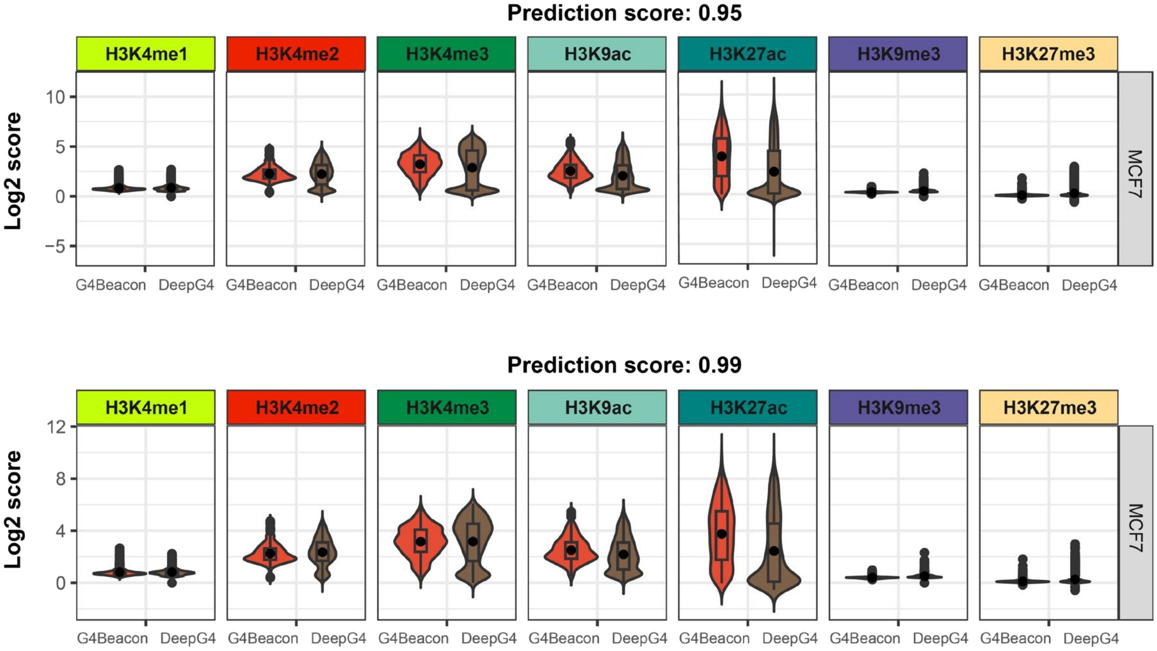


**Figure S11.** The boxplots of histone modification state scores of MCF7 active G4s derived from G4Beacon and DeepG4, with the probability thresholds of 0.95 and 0.99.

## Difference in Prediction Scores of Two-Tetrad G4s and Canonical Ones

In this research, we used G4-seq data as the candidate dataset. According to the research on G4-seq data, we knew that there were canonical G4-motifs as well as non-canonical ones (long loops, bulges, two-tetrads, etc.). To observe whether there was a difference in prediction performance between different motifs, we compared the canonical G4-motif result and the representative non-canonical G4-motif, two-tetrad result in the cross-cell-line experiment.

We used cannonical motif regex ( $G_{3-5}N_{1-7}G_{3-5}N_{1-7}G_{3-5}N_{1-7}G_{3-5}$ ) and two-tetrad motif regex ($G_2N_{1-7}G_2N_{1-7}G_2N_{1-7}G_2$) to assign canonical/two-tetrad tags on each G4-seq entry and filter out the entries with both cannonical and two-tetrad tags. We found that G4Beacon yielded better results in the canonical input situation. This result showed that the canonical in vivo G4s were more predictable when the sequence and chromatin accessibility were determined, while the two-tetrad in vivo G4 formation might be influenced by other environmental factors. This result was consistent with the basic truth that G4s with only two tetrads are more unstable.

**Table S5.** Cross-cell-line prediction results of different motif patterns (HepG2-trained model).

| | Accuracy | Precision | Recall | F1-Score | AUROC | AP |
|---|---|---|---|---|---|---|
| **K562-canonical** | 0.99 | 0.82 | 0.60 | 0.69 | 1.00 | 0.81 |
| **K562-2tetrads** | 0.99 | 0.76 | 0.66 | 0.71 | 1.00 | 0.78 |
| **MCF7-canonical** | 0.99 | 0.66 | 0.25 | 0.36 | 0.97 | 0.46 |
| **MCF7-2tetrads** | 0.99 | 0.50 | 0.20 | 0.28 | 0.97 | 0.32 |
